# Supplementary material for: A small dynamic leaf-level model predicting photosynthesis in greenhouse tomatoes
Source: PLoS One. 2023 Mar 16;18(3):e0275047. doi: 10.1371/journal.pone.0275047 (PMC10019686; doi:10.1371/journal.pone.0275047)
Supplement: S1 File — (PDF) [file pone.0275047.s001.pdf]

S1 File. A small dynamic leaf-level model predicting photosynthesis in greenhouse tomatoes

D Joubert [1 ¤* ◕], N Zhang [2 ‡], S.R. Berman [2 ‡], E Kaiser [2 ‡], J Molenaar [1 ‡], J.D. Stigter [1 ◕]

1 Mathematical and Statistical Methods Group, Wageningen University and Research, Wageningen, The Netherlands
2 Horticulture and Product Physiology, Wageningen University and Research, Wageningen, The Netherlands

◕These authors contributed equally to this work.
‡These authors also contributed equally to this work.
¤Current Address: Mathematical and Statistical Methods Group, Wageningen University and Research, P.O. Box 16, Wageningen, 6700AV, The Netherlands
* dominique.joubert@wur.nl

1 Definition of g_{tc} and calculating it from LICOR water vapour measurements

Broadly speaking air (in particular CO_2 and water vapour are important in this context) has to travel though 2 boundaries in order to reach the leaf mesophyll, 1) the boundary layer, a thin layer of calm air that surrounds each leaf, and 2) the stomata themselves. Since the device used to measure this state as output, LICOR, reports the conductance of water vapour, this is the starting point of our discussion.

The total resistance (r_t) to the flow of air into a leaf is therefore caused by the 2 boundaries in *series*. First, the resistance experienced due to the boundary layer (b) surrounding the leaf and then due to the stomatal opening itself (s). Resistance in series is computed by summating the 2 respective resistances and so the total resistance of the upper (u) leaf surface to the flow of *water vapour* (w) is:

$$r_{tw}(u) = r_{bw}(u) + r_{sw}(u) \tag{1.1}$$

Since flux simultaneously happens through both upper (u) and lower (l) surface, *in parallel*, the total resistance to the flow of water vapour is:

$$g_{tw} = \frac{1}{r_{tw}} = \frac{1}{r_{tw}(u)} + \frac{1}{r_{tw}(l)}, \tag{1.2}$$

$$= \frac{1}{r_{bw}(u) + r_{sw}(u)} + \frac{1}{r_{bw}(l) + r_{sw}(l)} \tag{1.3}$$

were g_{tw} denotes the total conductance of water vapour and the relationship between conductance and resistance is $conductance = \frac{1}{resistance}$. Describing the relationship between the lower and upper stomatal resistances as

$$K = \frac{r_{sw}(u)}{r_{sw}(l)} \tag{1.4}$$

and assuming the resistances on the upper and lower leaf surfaces to be equal, (1.3) can be written as

$$g_{tw} = \frac{1}{r_{bw} + (1+K)r_{sw}} + \frac{K}{Kr_{bw} + (1+K)r_{sw}}, \tag{1.5}$$

$$= \frac{1}{\frac{1}{g_{bw}} + (1+K)\frac{1}{g_{sw}}} + \frac{K}{K\frac{1}{g_{bw}} + (1+K)\frac{1}{g_{sw}}} \tag{1.6}$$

where g_{sw} and g_{bw} are the stomatal and boundary layer conductances of water respectively. Since we are modelling the pathway of carbon bound in the form of a CO_2 molecule, the total stomatal conductance to CO_2 diffusion (g_{tc}) is:

$$g_{tc} = \frac{1}{(1+K)\frac{1.6}{g_{sw}} + \frac{1.37}{g_{bw}}} + \frac{K}{(1+K)\frac{1.6}{g_{sw}} + K\frac{1.37}{g_{bw}}} \tag{1.7}$$

where 1.6 is the ratio of the diffusivity of CO_2 and H_2O in air, and 1.37 is the ratio of the diffusivity of CO_2 and H_2O in the boundary layer.

The LI-6400 LICOR uses a simplified version of this equation as:

$$g_{tc} = \frac{1}{\frac{1.6}{g_{sw}} - \frac{1.37(K^2+1)}{(K+1)^2 g_{bw}}} \tag{1.8}$$

Assuming K =1, (1.7) can be simplified to:

$$g_{tc} = \frac{1}{(1+1)\frac{1.6}{g_{sw}} + \frac{1.37}{g_{bw}}} + \frac{1}{(1+1)\frac{1.6}{g_{sw}} + 1\frac{1.37}{g_{bw}}} \tag{1.9}$$

$$g_{tc} = \frac{2}{(2)\frac{1.6}{g_{sw}} + \frac{1.37}{g_{bw}}} \tag{1.10}$$

This is the expression used to compute the measured stomatal conductance to CO_2 diffusion ($g_{tc,m}$) defined in (19) in the main text.

2 Definition of $G(I, C_a)$: Steady-state potential total leaf conductance to CO_2 diffusion obtainable as function of 2 environmental conditions

The function $G(I,C_a)$ defined in equations (15) and (16) can be interpreted as a steady-state target function describing the obtainable g_{tc} for a given set of environmental conditions. In the past this function has taken either: 1) irradiance (I) [1] or 2) leaf vapour pressure deficit (VPD_L) [2] into account. We expanded on this framework by taking ambient CO_2 concentration (c_a) into account.

We generated 2 sets of steady-state total stomatal CO_2 conductance data, one pertaining to c_a=400ppm and another to c_a=800ppm. For each set, the steady-state conductance was measured at 9 different light intensities: I=$\{0, 50, 100, 200, 400, 600, 800, 1000, 1200\}$, at an approximate leaf temperature (T_l) of $25°C$ and a relative humidity (RH) of 75%. The result is 2 parameterised functions:

$$G(I, c_a) = \begin{cases} \gamma_1(I, 400ppm) \\ \gamma_2(I, 800ppm) \end{cases} \tag{2.11}$$

each modelled as a standard saturation curve [1] for $i = 1, 2$ as:

$$\gamma_i(I) = g_{min} + \frac{\alpha I(g_{max} - g_{min})}{\sqrt{(g_{max} - g_{min})^2 + (\alpha I)^2}} \tag{2.12}$$

Fig.(2.1) is an example of the optimised function $\gamma_1(I, 400ppm)$ with $g_{min,400} = 0.1[molm^{-2}s^{-1}], g_{max,400} = 0.325[molm^{-2}s^{-1}]$ and $\alpha_{400} = 0.00033[mol\mu mol^{-1}]$. These parameter values are used for the model input $c_a \leq 500$. For $500 > c_a$, parameter values $g_{min,800} = 0.165[molm^{-2}s^{-1}]$, $g_{max,800} = 0.29[molm^{-2}s^{-1}]$ and $\alpha_{800} = 0.00017[mol\mu mol^{-1}]$ are used.

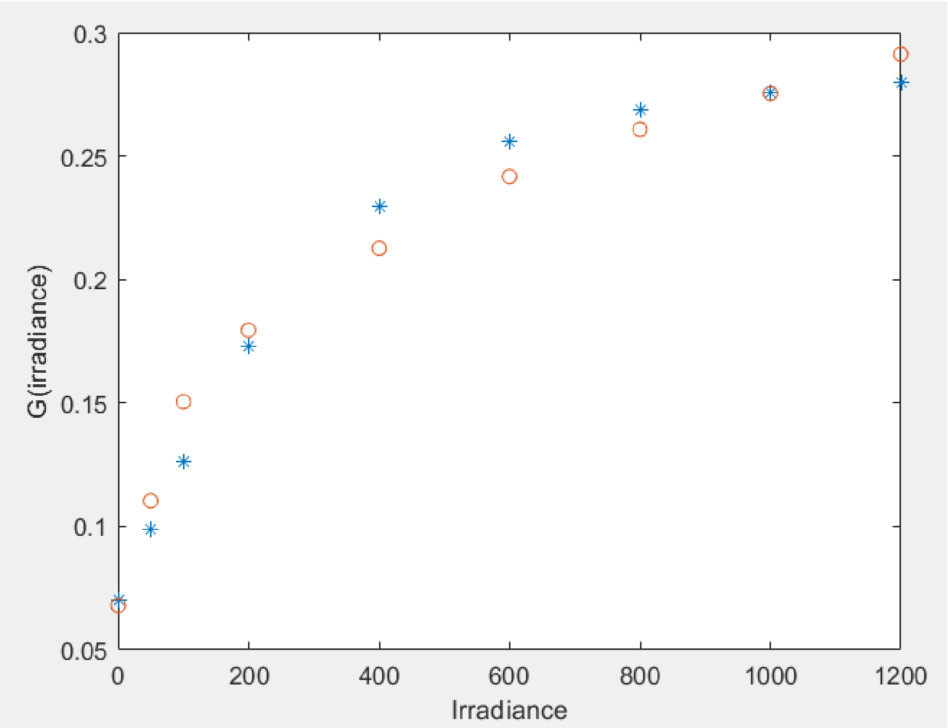

Fig 2.1. Optimised function G(I,400ppm). $g_{min,400} = 0.1$ $[molm^{-2}s^{-1}]$, $g_{max,400} = 0.325$ $[molm^{-2}s^{-1}]$ and $\alpha_{400} = 0.00033$ $[mol\mu mol^{-1}]$. * indicates predicted values for g_{tc} and o, measured values for g_{tc}.

3 Comparing model structures for stomatal conductance

We observed a 9% increase in model accuracy (expression (19)) when using 2 different time constants to describe stomatal conductance. The predicted differences are shown in Fig.(3.2).

We found no significant advantage in using expression (2.11), i.e. accounting for 2 different sets of ambient CO_2 concentrations. Using the data set measured under c_a=800ppm, the $RMSE = \sqrt{\sum (A_n - A_{n,m})^2/N}$ for results generated using G(I,400) and G(I,800) respectively, is given in Table (3.2).

Table 3.2. Comparing G(I,c_a) in (2.11). $RMSE = \sqrt{\sum (A_n - A_{n,m})^2/N})$ and R^2.

**Data** c_a**=800ppm RH=75%**	**Function** G(I,400)	G(I,800)
Validation set 5	0.90 [0.97]	0.89 [0.97]

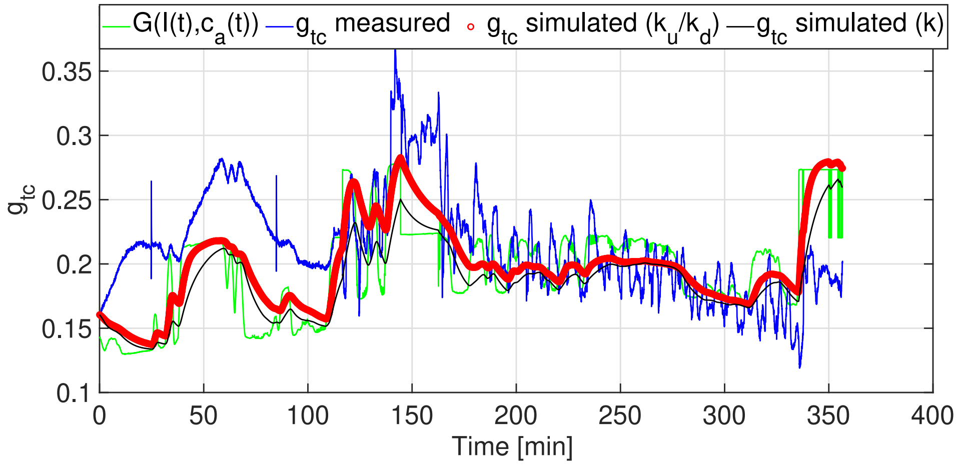

Fig 3.2. Comparing predictions for state g_{tc} by models that either assume symmetric (k) or asymmetric (k_u and k_d) stomatal responses to increases and decreases in irradiance Here $[k_u, k_d] = [179.4, 830.3][s]$ and $k = 401[s]$.

4 Modelling c_i using mass balance

The mass accumulation of CO_2 inside a leaf is described by a mass balance equation (see expression (18). It comprises a sink and 2 source terms. Two of the factors included in the model that limit c_i are: 1) **the opening/closing of stomata** (this is discussed in section 1.1). This is modelled as the amount of CO_2 conductance in the first ODE (see equations (15) and (16) and is included in the first CO_2 *source term* in (18). 2) **the activation/deactivation of C3 cycle enzymes** (discussed in section 1.2). This is described in the *sink term* in (18).

Here we give a brief derivation of the mass balance equation. The accumulation of a certain mass of CO_2 in a leaf over time is predicted by,

$$\begin{aligned}\frac{dm}{dt} =& (\text{m into leaf}) - (\text{m out of leaf}) \\ &+ (\text{m generated inside leaf}) \\ &- (\text{m consumed in leaf}) \end{aligned} \tag{4.13}$$

A mass balance can be expressed as a molar balance using the expression $n = MW \times \text{mass}$, where MW is the molecular weight of CO_2. Combining the molar flow of CO_2 into and out of the leaf and expressing it as the net molar flux, the molar balance is,

$$\begin{aligned}\frac{dn}{dt} =& (\text{n net flux}) + (\text{n generated inside leaf}) \\ &- (\text{n consumed in leaf}) \qquad [\frac{\mu mol}{s}] \end{aligned} \tag{4.14}$$

Multiplying (4.14) by $\frac{1}{n}$ allows us to predict the molar concentration,

$$\begin{aligned}\frac{d[n]}{dt} =& \frac{1}{n} \times [(\text{n net flux}) + (\text{n generated inside leaf}) \\ &- (\text{n consumed in leaf})] \quad [\frac{\frac{\mu mol}{mol}}{s}] \end{aligned} \tag{4.15}$$

Assuming that the ideal gas law, which relates pressure (P), volume (V), moles (n) and temperature (T) holds, $\frac{1}{n}$ can be written as:

$$\frac{1}{n} = \frac{RT}{PAd} \qquad [\frac{1}{mol} = \frac{\frac{m^3 Pa}{molK}K}{Pam^3}] \tag{4.16}$$

with leaf volume expressed here as V=Ad. Here (A) is the leaf area and (d) is the leaf thickness or the depth of inter-cellular airspace and photosynthetic tissue. We assume this to be constant [2].

Substituting (4.16) into (4.15) one obtains the general form of the molar balance often used ([2–4]):

$$\frac{d[n]}{dt} = \frac{RT}{PAd}[(\text{n net flux}) + (\text{n generated inside leaf}) - (\text{n consumed in leaf})] \quad [\frac{\frac{\mu mol}{mol}}{s}] \tag{4.17}$$

Comparing (18) to (4.17), we see that the first term in (4.17), the net molar flux, is Fick's law of diffusion. We use this as an approximation for the net photosynthetic rate (A_n). The second term, the molar concentration generated inside the leaf, is the mitochondrial respiration (R_d), modelled here as a constant value estimated *a priori* from steady-state data (see section 5).

Finally, the third term in (4.17), the molar concentration consumed in the leaf describes the activation/deactivation of C3 cycle enzymes. This is modelled by quantifying the availability (determined by the activation and deactivation) of the enzyme Rubisco and the limitation driven by the light dependent electron transport rate using the well-known FvCB model.

Notice that leaf area A is omitted from the pre-multiplier, this is due to the fact that stomatal conductance for example, is measured and modelled in units $[molm^{-2}s^{-1}]$. Accordingly, A [m^2] is omitted from (18).

Using the value defined by Noe as d = 0.1mm [2], the value of this pre-multiplier at a leaf temperature of $25°C$ is:

$$\frac{RT_l}{Pd} = \frac{8.314[\frac{m^3 Pa}{molK}]298.15[K]}{101325[Pa]0.00001[m]} = 2446.4 \ \ [\frac{m^2}{mol}] \tag{4.18}$$

5 Steady-state FvCB model parameter calculations

The steady-state parameters in (18) that need to be computed *a priori* are V_{cmax}, R_d and J(I(t)).

R_d - Mitochondrial respiration which includes CO_2 release in light other than photo-respiration

This value was estimated according to [5], as the y-intercept of the linear slopes of the A_n/c_i plot constructed using data at both low (50 $[\mu molm^{-2}s^{-1}]$) and high (1000 $[\mu molm^{-2}s^{-1}]$) irradiance. Data points were generated at 4 different c_a concentrations below 400ppm, RH=75% and $T_l = 25°C$. For the tomato this value was calculated as 1 $[\mu molm^{-2}s^{-1}]$. Fig.(5.3) shows an example of the measured data used to construct the graph in Fig.(5.4). To obtain R_d, the y-intercept between the 2 linear trendlines was computed. The data point at 50 $[\mu molm^{-2}s^{-1}]$ and 400ppm was omitted as an outlier in Fig.(5.3).

V_{cmax} - Maximum obtainable carboxylation rate

This parameter is computed from a steady-state A_n/c_i curve. The A_n/c_i measurements where made at T_l=24.7$°C$ and at a relative humidity of 68%. Light was

Ca	50PAR	Ci	An	1000PAR	Ci	An
50	last point i	55.54375	-1.19249		46.12589	0.529102
100		92.2972	0.458177		75.5965	2.774776
250		230.1303	1.484563		152.4639	9.674701
400		354.8056	1.58619		180.5438	11.56485

Fig 5.3. R_d is computed as the value of the y-intercept between 2 trendlines. One generated at 4 different c_a concentrations at 50 $[\frac{\mu mol}{m^2 s}]$ and another at 1000 $[\frac{\mu mol}{m^2 s}]$.

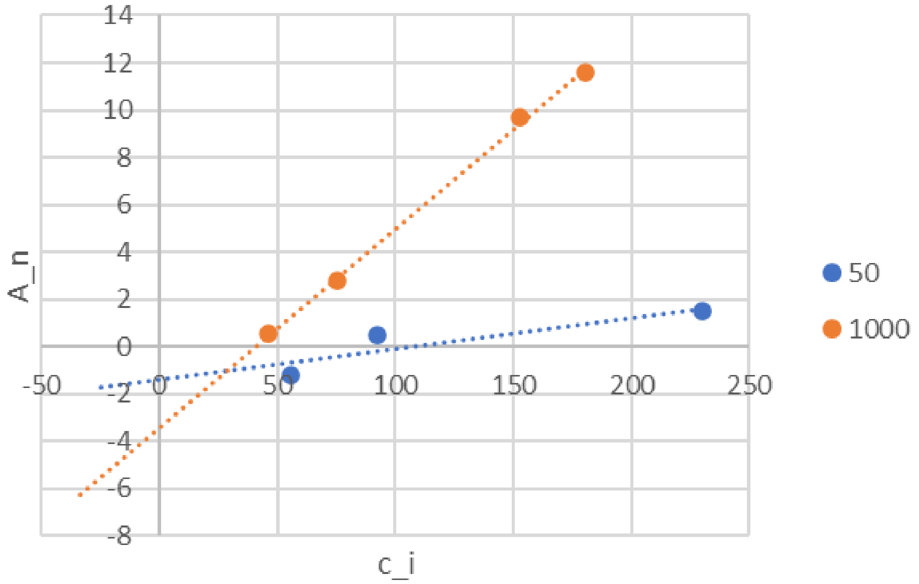

Fig 5.4. R_d is computed as the value of the y-intercept between 2 trendlines. One generated at 4 different c_a concentrations at 50 $[\frac{\mu mol}{m^2 s}]$ and another at 1000 $[\frac{\mu mol}{m^2 s}]$.

increased from a low light intensity (50 $[\mu mol m^{-2} s^{-1}]$) to 1800 $[\mu mol m^{-2} s^{-1}]$ in a single step response. This step change in light was done at 10 different ambient CO_2 concentrations [50, 100, 200, 300, 400, 500, 600, 800, 1000, 1200] $[\mu mol mol^{-1} s]$).

Steady-state A_n predictions were made using expression (7). Allowing for some re-arranging, the objective function used is:

$$O(V_{cmax}, J_{max}) \equiv min(\frac{(c_i - \Gamma^*(T_l))V_{cmax}}{(c_i + K_c(T_l)(1 + O_i/K_o(T_l)))}) - R_d, \frac{(c_i - \Gamma^*(T_l))J_{max}}{(4c_i + 8\Gamma^*(T_l))} - R_d) \tag{5.19}$$

The computed V_{cmax} is 99.25 $[\mu mol m^{-2} s^{-1}]$.

J(I) - Electron transport rate

Steady-state J values, measured at different irradiances were calculated using:

$$J(I)_{,m} = \frac{(A_{n,m} + R_d)(4c_i + 8\Gamma^*)}{(c_{i,m} - \Gamma^*)}, \tag{5.20}$$

and data generated at c_a=400ppm, RH=75%, and T_l=25$°C$. These measured values are indicated by ∘ in Fig.(5.6).

Modelled steady-state values for J(I) (indicated by * in Fig.(5.6)) was computed

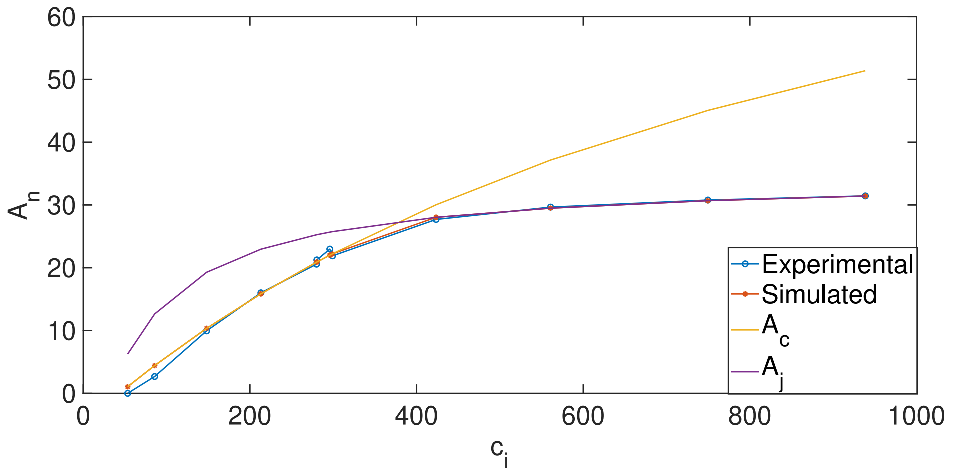

Fig 5.5. V_{cmax} is computed by optimising function in (5.19). Using R_d=1 $[\frac{\mu mol}{m^2 s}]$ and parameter values given in Table 3 in the main text.

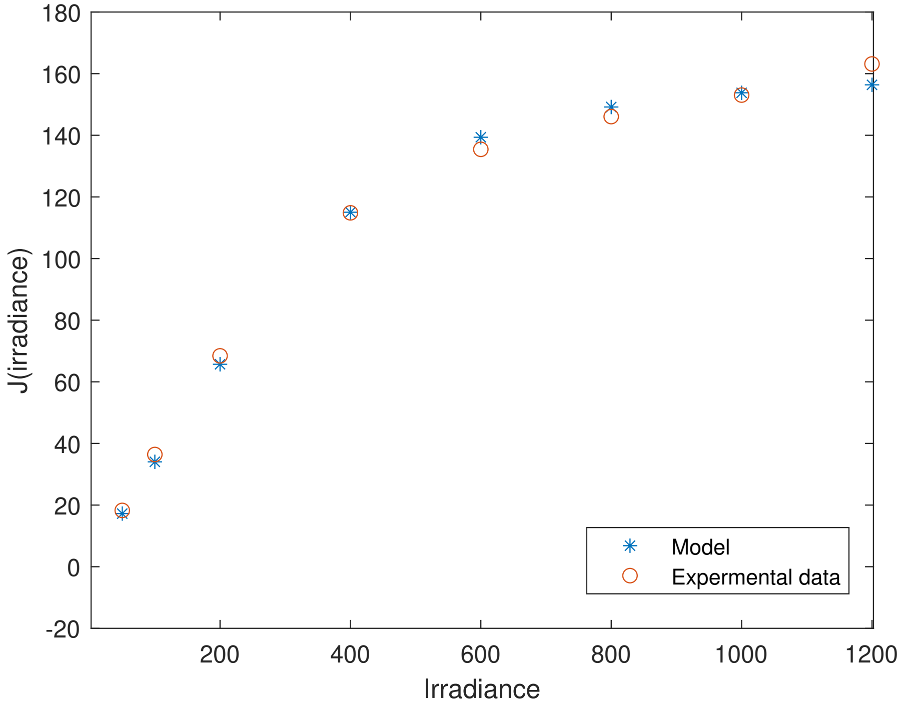

Fig 5.6. Measured steady-state J(I) values indicated by o (red). Modelled steady-state J(I) values indicated by * (blue). J_{max}=190.68, $\theta = 0.41$ and $\gamma = 0.9$.

using [6]:

$$J(I) = \frac{J_{max} + \theta I - \sqrt{(J_{max} + \theta I)^2 - 4J_{max}\gamma\theta I}}{2\gamma} \tag{5.21}$$

where J_{max} is the upper limit to potential chloroplast electron transport determined by the components of the chloroplast electron transport chain [7]. Parameters θ and γ are

unit-less.

6 Recipe of tomato nutrient solution used in this study

Available upon request from the author, dominique.joubert@wur.nl.

7 Matlab code and dynamic data

Available upon request from the author, dominique.joubert@wur.nl.

References

1. Vialet-Chabrand, S. and Dreyer, E. and Brendel, O. Performance of a new dynamic model for predicting diurnal time courses of stomatal conductance at the leaf level. Plant, Cell & Environment. 2013;36(8):1529-1546. doi:10.1111/pce.12086.

2. Noe SM, Giersch C. A simple dynamic model of photosynthesis in oak leaves: coupling leaf conductance and photosynthetic carbon fixation by a variable intracellular CO_2 pool. Funct Plant Biol. 2004;31(12):1195–1204. doi:10.1071/FP03251.

3. Vialet-Chabrand S, Matthews JSA, Brendel O, Blatt MR, Wang Y, Hills A, et al. Modelling water use efficiency in a dynamic environment: An example using Arabidopsis thaliana. Plant Science. 2016;251:65–74.

4. Morales A, Kaiser E, Yin X, Harbinson J, Molenaar J, Driever SM, et al. Dynamic modelling of limitations on improving leaf CO2 assimilation under fluctuating irradiance. Plant, Cell & Environment. 2018;41(3):589–604. doi:10.1111/pce.13119.

5. Laisk, A.K. Kinetics of photosynthesis and photorespiration of C3 in plants. Nauka Moscow. 1977.

6. Buckley, T. N, Diaz-Espejo, A. Reporting estimates of maximum potential electron transport rate. New Phytologist. 2015;205:14–17.

7. von Caemmerer S, Farquhar G, Berry J. In: Biochemical Model of C3 Photosynthesis. vol. 29; 2009. p. 209–230.
